# The Diseases of the Stomach

**Published:** 1900-03

**Authors:** 


**The Diseases of the Stomach.** By William W. Van Valzah, M.D., and J. Douglas Nisbet, M.D. Pp. 674. London: The Rebman Publishing Co., Ltd. 1899.

We think that the author has not altogether avoided the fault to which he alludes in his introduction as exemplified in many text-books, of describing "as distinct diseases what are in reality only the functional signs of disease," and of "the mistaking of a condition for a disease." He also carries the differentiation of diseases of the stomach to an excessive degree, beyond, in fact, the limits of a practical differential diagnosis. Thus, to take for example gastritis, no less than ten varieties are described, each with its appropriate group of symptoms, one of which is an old friend of the out-patient department, gastric fever; the latter surely a needless resuscitation in a scientific text-book. After this we read with relief that the author will not describe interstitial gastritis as a distinct form. The account of phlegmonous gastritis is insufficient in a special book of this kind, although it is such a rare disease. Then the author has given us a new nomenclature, no doubt very good, but the titles are too long; we take for example such a mouthful as adenohypersthenia gastrica, one variety of the "dynamic affections of secretion."

In places the book appears unpractical, as when, in the diagnosis of gastralgia, the differentiation from gastric ulcer instead of being given first as of the greatest importance, is put after a number of other affections.

We must also dissent from "anorexia nervosa" being treated in a work on diseases of the stomach as a gastric affection; such a view leads to a totally erroneous idea of the nature of the disease and of its proper treatment. In spite, however, of the above unfavourable remarks which we have felt obliged to make and have put first, there is a good deal to praise in the book, and it will be found to contain a quantity of information by anyone who can give time to its study. There is much common sense in the advice on treatment. Unfortunately, owing to the too diffuse style in which it is written, it is of too great length, and there is a good deal of irrelevant writing which obscures the real merit of the work; for it is a book of real value, and obviously the outcome of much knowledge of the subject. Especially we recommend the very full section on the methods of clinical investigation of diseases of the stomach, which will be found most useful for reference. The advice as to the methods to be employed and the treatment to be carried out is trustworthy and good, and the book is a mine of information on these points and on the composition of articles of food and matters of diet generally. In advice as to diet the author errs, perhaps, rather on the side of over-detail; but this can hardly be called a fault in a book written for medical men.

